# Unveiling the power of flavonoids: A dynamic exploration of their impact on cancer through matrix metalloproteinases regulation

**DOI:** 10.37796/2211-8039.1447

**Published:** 2024-06-01

**Authors:** Peramaiyan Rajendran

**Affiliations:** a Department of Biological Sciences, College of Science, King Faisal University, Al Ahsa, 31982, Saudi Arabia; b Centre of Molecular Medicine and Diagnostics (COMManD), Department of Biochemistry, Saveetha Dental College & Hospitals, Saveetha Institute of Medical and Technical Sciences, Saveetha University, Chennai 600 077, Tamil Nadu, India

**Keywords:** MMP, Metastasis, Flavonoids, Apigenin, Naringin, Kaempferol, Malvidin, Genistein

## Abstract

Cancer stands as a significant contributor to global mortality rates, primarily driven by its progression and widespread dissemination. Despite notable strides in cancer therapy, the efficacy of current treatment strategies is compromised due to their inherent toxicity and the emergence of chemoresistance. Consequently, there is a critical need to evaluate alternative therapeutic approaches, with natural compounds emerging as promising candidates, showcasing demonstrated anticancer capabilities in various research models. This review manuscript presents a comprehensive examination of the regulatory mechanisms governing the expression of matrix metalloproteinases (MMPs) and delves into the potential therapeutic role of flavonoids as agents exhibiting specific anticancer activity against MMPs. The primary aim of this study is to elucidate the diverse functions associated with MMP production in cancer and to investigate the potential of flavonoids in modulating MMP expression to inhibit metastasis.

## 1. Introduction

Cancer is a prominent contributor to global disease burden and mortality. Consequently, over the past twenty years, extensive biomedical research has produced a vast quantity of knowledge on the molecular processes involved in the development of cancer and the signaling pathways that contribute to its growth. The intricate interactions between tumor cells and the tumor microenvironment are crucial in determining the outcome of this process [1]. Research undertaken for over four decades has consistently shown increasing evidence that extracellular matrix remodeling proteinases, namely matrix metalloproteinases (MMPs), play a crucial role in the changes observed in the microenvironment throughout the evolution of cancer [2]. They also have a role in pathological situations like inflammatory, vascular, and autoimmune disorders, as well as carcinogenesis. MMPs have been regarded as promising diagnostic and prognostic biomarkers in many forms and phases of cancer. Natural products have emerged as promising contenders in cancer research, offering a rich source of bioactive compounds with diverse therapeutic potential. Among these, flavonoids stand out for their remarkable properties [3]. Extensively found in fruits, vegetables, and other plant sources, flavonoids have garnered attention for their anti-cancer effects, particularly in modulating key processes such as matrix metalloproteinases expression. Their multifaceted actions make them valuable candidates for further exploration in cancer treatment, providing a natural and holistic approach to combat this complex disease [4]. The ongoing research into the impact of natural products, including flavonoids, holds great promise for the development of novel and effective strategies in the fight against cancer.

MMPs, belong to the extensive metzincin super-family, which includes other enzyme families such as astacins, serralysins, reprolysins, and adamalysins metalloproteinases [5,6]. Traditionally, MMPs were thought to have a primary role in degrading various elements of the extracellular matrix (ECM) and the basement membrane, primarily associated with tissue remodeling and maintenance. However, recent investigations into MMP substrates have unveiled a more complex picture. These enzymes are now recognized for their involvement in the regulation of the release or activation of crucial molecules such as chemokines, cytokines, growth factors, antimicrobial peptides, and other bioactive compounds [7,8]. Consequently, MMPs contribute significantly to a broader range of physiological processes, including innate and adaptive immunity, inflammatory responses, angiogenesis, bone remodeling, and the growth of neurites [7,9–12].

MMPs play a pivotal role in cancer metastasis, which is the process by which cancer cells spread from the primary tumor to distant organs or tissues, contributing significantly to the lethality of many cancer types [13–16]. MMPs are a family of enzymes that are responsible for the degradation and remodeling of the ECM, a crucial scaffold of proteins that surrounds cells. In the classical view, MMPs were primarily regarded as tissue remodeling enzymes, but their influence on cancer metastasis has become increasingly evident [11,12,14,17,18]. Cancer cells exploit MMPs to facilitate their metastatic journey in several ways. Firstly, MMPs assist cancer cells in breaking down the ECM, creating pathways through which they can invade nearby tissues and blood vessels. They degrade components of the basement membrane, which is a barrier that separates tumor cells from the bloodstream. This allows cancer cells to intravasate into the bloodstream or lymphatic system, enabling them to travel to distant sites in the body. Moreover, MMPs can activate proinflammatory cytokines and chemokines, which in turn attract immune cells to the tumor microenvironment. These immune cells may create an environment that supports cancer cell survival and further migration. Additionally, MMPs are implicated in angiogenesis, the formation of new blood vessels, which is essential for providing oxygen and nutrients to growing tumors. The overexpression of specific MMPs is often associated with increased metastatic potential in various cancer types. Elevated MMP levels can disrupt the delicate balance between ECM synthesis and degradation, promoting tumor invasion and facilitating metastasis [19]. This makes MMPs attractive targets for potential therapeutic interventions to impede cancer metastasis. While the role of MMPs in cancer metastasis is well-established, the complexity of their functions and the various factors influencing their activity make them subjects of ongoing research. Targeted therapies and inhibitors are under development to selectively block MMP activity and, in turn, hamper metastatic progression, potentially providing new avenues for cancer treatment and improving patient outcomes. Understanding the intricate interplay between MMPs and cancer metastasis is crucial for advancing our knowledge of the disease and developing effective strategies for its control and management.

Flavonoids, a diverse group of natural compounds found abundantly in fruits, vegetables, and plant-based foods, have garnered significant attention for their potential role in cancer metastasis [20–23]. Emerging evidence suggests that flavonoids may exert a range of effects that can influence the various stages of the metastatic process in cancer [24–26]. Flavonoids are known for their antioxidant and antiinflammatory properties, which can help counteract the cellular processes involved in tumor invasion, migration, and angiogenesis [27–32]. These compounds have been shown to suppress the activity of MMPs, enzymes critical in degrading the extracellular matrix and facilitating tumor cell dissemination. Furthermore, flavonoids have the potential to modulate key signaling pathways involved in cancer metastasis, influencing the expression of genes related to cell adhesion, motility [33–35]. In addition to their direct effects on tumor cells, flavonoids have demonstrated the ability to regulate the tumor microenvironment, creating an inhospitable terrain for cancer cells to establish secondary growths [36]. While the body of research on flavonoids and their antimetastatic properties is expanding, more investigations are needed to elucidate the specific mechanisms of action, optimal dosages, and potential therapeutic applications. This comprehensive review compiles a range of preclinical investigations that underscore the impact of flavonoids on matrix metalloproteinases (MMPs) in the context of cancer metastasis. We delve into the intricate cellular mechanisms involved and provide insights from pre-clinical studies, underscoring the potential of flavonoids in mitigating metastasis, both as a standalone therapeutic approach and in conjunction with conventional treatments, with the aim of augmenting the overall effectiveness of cancer management for patients.

## 2. Methods

The objective of this review is to explore and synthesize the anti-cancer attributes of flavonoids, with particular attention to their influence on critical stages of cancer advancement, encompassing cancer cell migration, invasiveness, and the creation of metastatic growths. This comprehensive review aims to consolidate existing knowledge regarding the effectiveness of flavonoids in impeding cancer progression by analyzing both preclinical and clinical research in the field of oncology. Given the encouraging outcomes observed in laboratory and animal studies, this review underscores the significance of integrating flavonoids into clinical investigations. This strategy underscores a precise and individualized approach to anti-cancer therapy. The primary data source for this review was the biomedical literature, primarily acquired through searches within the PubMed database. The search terms encompassed “metastasis” and various subcategories of flavonoids, including “flavonoids,” “flavanones,” “flavonols,” “flavones,” “flavanols,” “isoflavonoids,” “chalcones,” “anthocyanidins,” and related terminology. Furthermore, this review particularly spotlights recent scientific articles published between 2015 and 2023 to ensure the currency and relevance of the data and insights pertaining to the latest developments in the realm of flavonoid research and their potential anti-cancer attributes.

## 3. Structure and functions of MMPs

To date, researchers have identified and characterized at least 25 distinct MMPs in vertebrates [37]. Within the human genome, there are 24 different MMPs, including two identical forms of MMP-23, which are encoded by two separate genes, known as MMP23A and MMP23B [38]. The remarkable diversity observed in today’s mammalian MMP gene families primarily arises from an extensive history of gene tandem duplication and exon shuffling that occurred during the course of evolution among tetrapods. It is important to note that some of the existing MMP members are likely derived from a single ancestral gene, leading to the formation of a cluster of MMP genes [38]. The primary role of MMPs, has long been associated with the degradation and clearance of ECM components within tissues, it is increasingly evident that this enzymatic activity extends to modifying crucial cell–matrix and cell–cell interactions [39]. Table 1 provides illustrative instances of MMP actions that hold the potential to influence diverse cellular processes, including cell migration, differentiation, proliferation, inflammatory responses, angiogenesis, and apoptosis [40]. It has been reported that MMP-2 is present intracellularly within cardiac myocytes, as well as co-localizing with troponin I in cardiac myofilaments in several studies [41]. Nuclear extracts from the heart and liver of rats have been found to contain MMP-2 activity [42]. MMP2 is capable of cleaving poly ADP-ribose polymerase, an enzyme integral to DNA repair processes [43]. MMP inhibitors have shown to effectively inhibit this cleavage in in vitro studies. MMP-2’s presence within the nucleus suggests it may play an important role in poly ADP-ribose polymerase degradation, which may have implications for DNA repair [44,45].

**Fig. 1:**
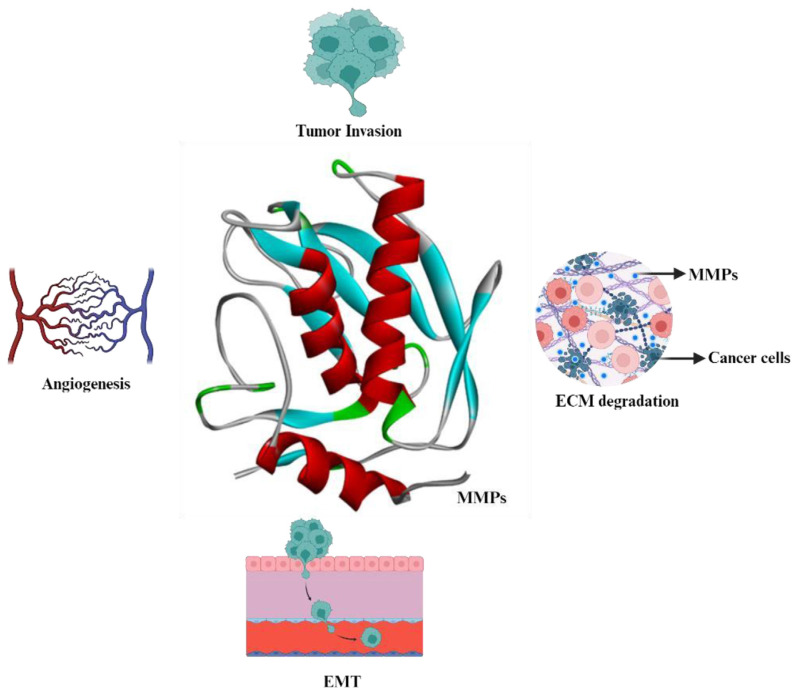
MMPs role in cancer.

This unique organization of MMP genes has been conserved from amphibians through to mammals, underscoring its evolutionary significance. Among these, considerable attention has been directed towards MMP-2 and MMP-9 (Fig. 1), recognized as gelatinases for their ability to degrade type IV collagen, a prominent constituent of basement membranes that serve as a crucial barrier separating epithelial cells from the underlying stromal tissue [64]. The elevated expression and activity of MMP-2 and MMP-9 in tumor contexts instigate the breakdown of these basement membranes, a pivotal step in tumor invasion and the metastatic process [6].

## 4. Molecular mechanism of MMPs on cancer metastasis

Among the 25 recognized MMPs, several MMPs have been associated with the development and advancement of cancer [65]. Elevated expression of MMP1, MMP2, MMP3, MMP7, MMP9, MMP13, and MMP14 has been consistently associated with tumor progression, metastasis, and a less favorable prognosis. MMP9, for instance, has demonstrated a critical role in driving tumor progression and metastasis, notably in triple-negative breast cancer and the early stages of melanoma [14,66]. Research has unveiled mixed findings regarding MMP9 expression in relation to breast and colon cancer survival rates, indicating its multifaceted impact on cancer outcomes [67–69]. Notably, studies involving mice deficient in MMP2 or MMP9 have underscored their essential role in promoting colonization and tumor proliferation [70]. Additionally, overexpression of MMP3, MMP13 and MMP14 has been linked to the promotion of mammary carcinogenesis [71,72]. In the context of colon cancer, survival rates have shown correlations with MMP12 expression [73,74]. These findings collectively emphasize the intricate roles played by various MMPs in cancer growth and metastasis. Importantly, the expression levels and functions of MMPs are influenced by the specific cancer stage and type, underscoring the complex relationship between MMPs and cancer progression.

In numerous cancer types, MMPs are expressed; however, the key to assessing tumor metastatic potential lies in the levels of activated MMPs rather than their total presence. Post-transcriptional regulation of MMP activity is governed by two primary mechanisms: the activation of latent precursors, or zymogens, and the inhibition of active enzymes by tissue inhibitors of MMPs, also known as tissue inhibitors of metalloproteinases (TIMPs). Inactive proenzyme MMPs are secreted and are subsequently activated extracellularly, and it’s noteworthy that some MMPs can activate each other. For instance, MMP1 and MMP14 can jointly activate MMP2. The catalytic site of MMPs, particularly the zinc ion, is subject to regulation by TIMPs. TIMP1 and TIMP2 are recognized as the most versatile inhibitors, effectively blocking the activity of most MMPs. Notably, studies in mice have demonstrated that elevating TIMP1 levels can diminish the occurrence of brain and liver metastasis [75].

**Fig. 2:**
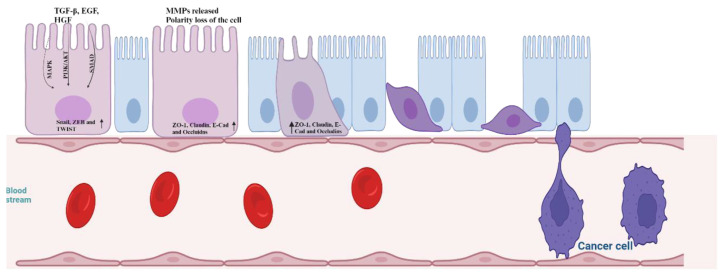
MMPs on Epithelial Mesenchymal Transition (Image created from Biorender dated 19.10.2023).

Upregulation of MT1-MMP expression has been shown to enhance metastasis, predominantly through the induction of epithelial-to-mesenchymal transition (EMT) [76,77]. This transition is marked by the downregulation of E-cadherin (E-Cad) and the concurrent upregulation of transcription factors such as TWIST, ZEB, and ZEP-1 in squamous cell carcinoma [78–80]. EMT programs in cancer are activated by various signaling molecules, including TGF-β, epidermal growth factor (EGF), and Hepatocyte growth factor (HGF) [81–84]. Cadherins, which are transmembrane glycoproteins facilitating cell–cell adhesion, not only support these connections but also help maintain normal tissue architecture. In cancer, different cadherins serve diverse roles in tumor development. A notable occurrence during EMT is the cadherin-switch, where E-Cad is lost, and N-Cadherin (N-Cad) is expressed [85,86]. This switch can induce or intensify the metastatic potential of tumor cells. E-Cadherin, by preventing the dissociation between cells within the tumor mass, functions as a barrier to inhibit the spread of cancer to other tissues. The loss of E-Cadherin can also lead to the mislocalization of β-catenin and p120 catenin, subsequently triggering MAPK activation [87]. E-Cad operates as a tumor-suppressor protein, significantly influencing cancer progression [88]. The transition from E-Cad to N-Cad in the cadherin switch is tightly regulated by signaling pathways like Wnt and TGF-b, orchestrating the downregulation of E-Cad while inducing the expression of N-Cad. This molecular shift triggers N-Cad to activate cell proliferation through PI3K/AKT, SMAD and MAPK pathways [80,84,89–91]. MMPs also play a pivotal role in epithelial-to-mesenchymal transition (EMT) through various mechanisms (Fig. 2). EMT-prone cells tend to produce higher quantities of MMPs, facilitating invasion and metastasis. Among the MMPs implicated in EMT, MMP1, MMP2, MMP3, MMP7, MMP9, MMP14, and MMP28 are the prominent contributors [92]. During EMT, cells acquiring a stromal-like phenotype further advance cancer progression by promoting additional MMP production [93]. This intricate interplay of MMP activity is a fundamental feature in both normal physiological processes and pathological conditions, and its regulation hinges on diverse mechanisms.

## 5. Flavonoids on cancer metastasis

Cancer therapeutic goals include both preventing metastasis in high-risk patients and preventing additional metastases. Metastasis’ biological heterogeneity is a major obstacle to treatment. Failure in any of these steps can prevent metastasis. MMPs play a key role in cancer formation and metastatic niche establishment in secondary organs. Cell adhesion and cytoskeleton proteins, chemokines, growth factors and ECM degradation play a crucial role in cancer progression.

**Fig. 3:**
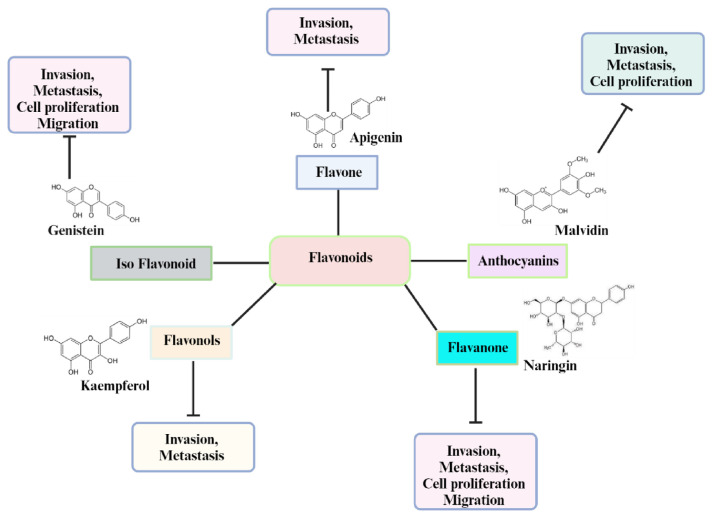
The chemical structure of flavonoids that regulate the expression of MMPs.

Nonetheless, the characteristics of metastatic tumors diverge from those of the initial parent cells. Metastasis signifies the emergence of a resilient and invasive subset of the primary tumor, marked by additional genetic or epigenetic modifications, often compounded by previous therapeutic interventions. In several studies, flavonoids have been shown to suppress the metastasis of various types of cancer [94–98] (Fig. 3).

**Fig. 4:**
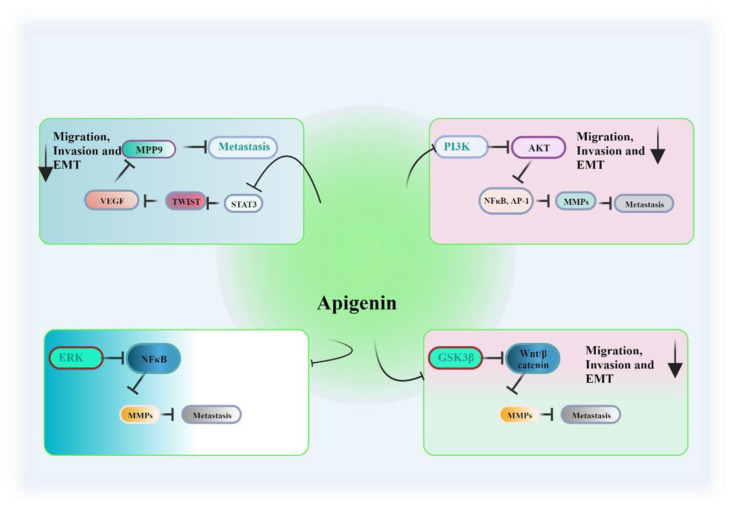
Signaling pathways regulating MMPs by Flavone Apigenin.

Plants flavonoids as a secondary metabolic product, consisting of 15-carbon phenylpropanoid chains linked by a heterocyclic pyran ring [20,99,100]. Among the most common natural compounds found in cereals, fruits, stems, herbs, nuts and flowers these are responsible for flowers’ color and fragrance [101]. There are six basic types of flavonoids: flavonols, flavanones, isoflavonoids, anthocyanidins, flavones and flavanols [21]. Flavonoids have been shown to inhibit cancer, invasion, and metastasis in numerous in vitro and in vivo studies (Fig. 3, regulating apoptosis, ECM remodeling, EMT, oxidative stress and inflammation [22,23,102]. Cancer-related signaling pathways such as MAPK, Akt, JNK, NF-kB, and growth factors like VEGF, cytokines, and chemokines can be modulated by flavonoids [36,103–106] (see Fig. 4).

### 5.1. Apigenin on MMPs regulation

Numerous vegetables, among them tarragon, celery, parsley, onions, and more, are known to contain apigenin. In its natural state, apigenin is commonly found in the form of glucosides, including 7-O-glucosides and 6- or 8-C-glucosides [107]. Importantly, apigenin can be converted into its free form following ingestion. This property raised substantial safety concerns, as well as the intriguing capability of apigenin to differentiate between normal cells and cancerous ones [108]. Apigenin is capable of modulating several key mediators involved in the process of metastasis. Notably, it can effectively inhibit STAT3, a protein associated with the promotion of angiogenesis, resistance to apoptosis, and the upregulation of MMP2, MMP-9, and Twist1 [109]. By curtailing the phosphorylation of STAT3 and the subsequent expression of downstream genes like MMP-2 and MMP-9, apigenin exerts a suppressive effect on the invasion and migration of cancer cells [110]. Both in vitro and in vivo studies have demonstrated that apigenin’s inhibitory action on STAT3 is more pronounced in hepatocellular carcinoma cells compared to normal liver cells, leading to reduced migration and metastatic markers [111]. Apigenin’s interference with metastasis is multifaceted, encompassing the inhibition of various kinases, such as MAPKs, and the suppression of the IGF/IGFBP-3 pathways [112,113]. Within lung and colon carcinoma cells, apigenin exhibits the potential to enhance the expression of anti-meta-static proteins, including CD26 [114,115]. Additionally, its role in inhibiting metastasis extends to the suppression of the PI3K/Akt pathway. This pathway is known to be activated by HGF, which can trigger invasion and metastasis [116]. Specifically, HGF induces cell–matrix adhesion through the PI3K pathway. Apigenin intervenes by inhibiting the phosphorylation of PI3K/Akt, thereby mitigating these alterations in MDA-MB231 breast cancer cells. Furthermore, apigenin’s inhibitory effect on Akt contributes to the downregulation of MMP-9, a pivotal driver of malignant metastasis [116,117].

**Fig. 5:**
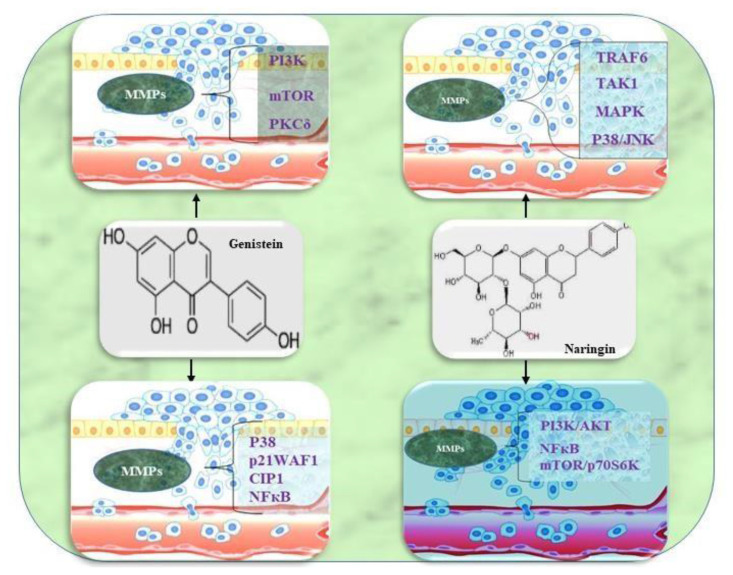
Signaling pathways regulating MMPs by Genistein and Naringin.

Research investigating the impact of apigenin on the MMP/uPA/TIMP protein family in gastrointestinal cancers remains relatively limited in this realm of cellular regulation. Nevertheless, flavonoids like apigenin have demonstrated the capacity to induce epithelial differentiation and modulate cell migration in colon epithelial cells, potentially involving MMPs [118]. Although a portion of these effects has been primarily observed in breast cancer cells, apigenin has shown the ability to inhibit uPA mRNA production and protein secretion while leaving uPA receptor levels unaffected [115,118]. Additionally, it effectively suppresses the expression of MMP-9 triggered by both phorbol 12-myristate 13-acetate and epidermal growth factor, alongside basal MMP- 9 production. There is evidence to suggest that apigenin effectively impedes the metastasis of OVCAR-3 cells by targeting MMP2, MMP9 through the PI3K/AKT/mTOR/p70S6K1 signaling pathways [119]. In Western blotting experiments, it was observed that apigenin can also lead to a reduction in MMP-2 expression. Furthermore, various studies have indicated that apigenin exerts its inhibitory effects on MMPs activity in diverse cell types by modulating the ERK MAPK pathway [120,121]. In in vitro cell studies, apigenin has demonstrated its ability to inhibit tumor growth through various pathways, encompassing NF–B, JNK, and ERK pathways. These pathways are believed to play a role in apigenin’s induction of apoptosis in MDA-MB- 231 cells. Furthermore, apigenin, by reestablishing the equilibrium between MMP and its counterpart TIMP, effectively hinders the metastasis of breast cancer. Exposure to apigenin has been shown to lead to a decrease in proliferation and the expression of genes associated with invasion and metastasis in human pancreatic cancer cells BxPC-3 and PANC-1 [122,123]. This effect is notable in the reduction of MMP-1, MMP-2, and MMP-9 activity, lowered mRNA levels of KRAS and GSK3, and an increase in TIMP-1 levels.

he canonical Wnt pathway has been shown to promote epithelial–mesenchymal transition (EMT) through multiple mechanisms. Wnt/beta-catenin signaling upregulates various mesenchymal markers, such as SLUG, ZEB1, and TWIST, which in turn repress E-cadherin and elevate MMPs. MMP-3, by cleaving the ectodomain of E-cadherin, remodels the extracellular matrix, enhancing invasion while inactivating E-cadherin [124]. Additionally, Wnt signaling can upregulate SNAIL through the inhibition of GSK3B phosphorylation and SNAIL degradation, contributing to EMT [125]. This WNT pathway activation has been associated with increased cancer metastasis. In a study aimed at assessing whether osteosarcoma progression could be disrupted through apigenin consumption by inhibiting the Wnt/beta-catenin pathway, apigenin was found to suppress both the intrinsic and activated transcriptional activities of beta-catenin/TGF, significantly reducing its nuclear concentration [126,127] (Fig. 5). Activation of MMP9, by contrast, was observed to inhibit osteosarcoma invasiveness by downregulating Wnt/beta-catenin signaling. Apigenin has been shown to inhibit MMPs activity and expression in various cancer cell types, including breast, prostate, and melanoma. By doing so, apigenin can impede the invasive capabilities of cancer cells and reduce their potential to metastasize. This promising attribute of apigenin underscores its potential as an anti-metastatic agent in the fight against cancer.

#### 5.1.1. Malvidin on MMPs regulation

Malvidin stands out as the primary anthocyanin monomer within blueberry anthocyanins. When administered to Huh-7 cells, malvidin yielded several notable effects: it inhibited cell proliferation and the cells’ ability to form colonies, induced cell cycle arrest, and triggered apoptosis in a dose-dependent manner. Additionally, through the regulation of MMPs expression, malvidin effectively curtailed the migration and invasion potential of Huh-7 cells. In summation, these findings underscore the anti-hepatocellular carcinoma (HCC) potential of malvidin, which is associated with its inhibition of MAPK, Akt/PTEN, and MMP pathways [128]. Malvidin were also found to exert an inhibitory effect on cellular migration, as evidenced by wound healing and Boyden chamber assays. In the intricate process of cancer cell metastasis, matrix-degrading proteinases play a crucial role. Notably, treatment of B16–F1 cells with varying concentrations of malvidin resulted in a reduction of ECM proteinases, specifically MMP-2 and MMP-9, as demonstrated by gelatin zymography assays. Further insights from Western blotting assays revealed diminished expression levels of key proteins, including Ras, PI3K/AKT, and NF-κB in malvidin-treated B16–F1 cells. This collective evidence suggests that malvidins have the potential to modulate B16–F1 cell metastasis by suppressing MMP-2 and MMP-9 activities through the inhibition of the Ras/PI3K signaling pathway. Additionally, B16–F1 melanoma cells were inoculated into the right groin of C57BL/6 mice, who were concurrently administered malvidin in their diet [129]. Malvidin effectively curbed the proliferation, polarization, migration, and invasive potential of HepG2 cells by modulating the protein expression of key markers. This included the downregulation of cyclin D1, cyclin B, and cyclin E, as well as an upregulation of caspase-3, cleaved caspase-3, and Bax. Furthermore, it activated phosphatase and tensin homologue deleted on chromosome 10 (PTEN), concomitant with a reduction in p-AKT levels. Importantly, Malvidin also attenuated the protein expression of MMP-2 and MMP-9 [130].

### 5.2. Regulation of MMPs by flavanone naringin

Among the flavonoids found in citrus fruit, naringin emerges as a prominent constituent. Findings from animal studies suggest that naringin boasts anticancer, antioxidative, and anti-atherogenic properties [131–133]. Notably, in the context of U251 glioma cells, naringin exhibited inhibitory effects on invasion and migration across various concentrations. It also led to a decrease in MMP-2 and MMP-9 expression, as well as a reduction in proteinase activity [134,135]. Simultaneously, the expressions of TIMP-1 and TIMP-2 increased. The phosphorylation of p38 was significantly attenuated by naringin treatment. Furthermore, when combined with SB203580 and MMP-2 and MMP-9 inhibitors, it resulted in a synergistic reduction in MMP-2 and MMP-9 expression and an increase in TIMP-1 and TIMP-2 expressions [135,136]. In U87 cells, naringin attenuated MAPK signaling pathways, including ERK, JNK, and p38, ultimately leading to the downregulation of MMP-2 and MMP-9 expression and their associated activity [135,137]. These findings underscore the potential utility of naringin as an anti-metastatic agent. Exposure of pancreatic cancer cells, specifically aspc-1 and panc-1, to TGFβ1 triggered distinct changes in their characteristics. This included notable alterations in their EMT morphology, increased cell motility, and the development of resistance to gemcitabine. These changes were accompanied by an elevation in the expression of EMT markers, such as vimentin, N-cadherin, MMP2, and MMP9 [138–140]. Importantly, Naringin intervened by inhibiting the TGF-β1/Smad3 signaling pathway, effectively curbing both the mRNA and protein expression of EMT markers in these pancreatic cancer cells [141]. Consequently, naringin not only suppressed their migration and invasion capabilities but also reversed their acquired resistance to gemcitabine [142]. In TNF-α-induced VSMC, treatment with the aglycone naringenin yielded comparable outcomes to naringin [143]. These included similar levels of MMP-9 expression, invasion, migration, and AKT phosphorylation. Naringin effectively inhibited TNF-αinduced VSMC by targeting the PI3K/AKT/mTOR/p70S6K pathway, leading to the repression of invasion, migration, and MMP-9 expression [104]. Additionally, MMP-9 expression was suppressed through the modulation of NF-kB and activator protein-1 [144]. Naringin’s efficacy in blocking Smad3 and Smad7 signaling within the TGF-β-rich tumor microenvironment was demonstrated in mouse models of melanoma and lung carcinoma [145]. This inhibition resulted in a significant suppression of tumor invasion and metastasis. Naringin effectively targeted the NF-kB and MT1-MMP axis, thereby inhibiting Smad3-mediated MMP2 transcription and concurrently increasing TIMP levels [146]. Furthermore, Naringin boosted Smad7 expression, leading to the suppression of TGF-β/Smad3 signaling while activating MMP2. The combined action of naringin effectively thwarted melanoma invasion and metastasis by targeting MMP2 at multiple levels, encompassing transcription, post-translational activation, and function, all through the modulation of the TGF-β/Smad pathway. Naringin has been shown to inhibit MMP expression and activity in various cancer cell lines, including glioma, glioblastoma, and breast cancer. This action of naringin hinders the ability of cancer cells to invade surrounding tissues and metastasize, making it a potential candidate for anti-metastatic therapies in cancer treatment.

### 5.3. Flavonols (kaempferol) on MMPs regulation

Tea, grapes, broccoli, and berries contain high levels of kaempferol, a naturally occurring polyphenol in the flavonoid group. Kaempferol has been found to have antioxidant, anti-inflammation, and antitumor properties in several previous studies [3,147]. Human ovarian cancer cells were found to be susceptible to kaempferol inhibitory effects on angiogenesis and VEGF expression. Human glioma and leukemia cells were also apoptotic by kaempferol through AKT expression. According to recent studies, kaempferol inhibits the G2/M cell cycle in human hepatic cancer cells and induces autophagic cell death. Lin et al. reported that kaempferol exerts its down-regulatory effect on MMP-2 by inhibiting ERK1/2 phosphorylation and activating AP-1 [148]. Additionally, in another study, it was found that kaempferol significantly reduced the phosphorylation of AKT, while leaving the MAPK pathway unaffected. These observations suggest that kaempferol diminishes AKT activation, which may play a role in the expression of MMP-9 and cell invasion. Collectively, these findings underscore the potent antimetastatic activity of kaempferol in HCC cells [149]. Kaempferol demonstrates a remarkable ability to curb the invasion and migration of 786-O renal cell carcinoma (RCC) cells without exerting any cytotoxic effects. To decipher the underlying mechanisms responsible for its anti-invasive properties, comprehensive Western blot analyses were conducted. The results revealed that kaempferol effectively mitigates the expression and activity of MMP-2. This inhibitory effect on MMP-2 can be attributed to the downregulation of Akt and focal adhesion kinase (FAK) phosphorylation [150]. Remarkably, research findings have illuminated kaempferol capacity to impede the proliferation of liver cancer cells. This inhibitory action is achieved by activating mitochondrial signaling pathways, while simultaneously suppressing migration and invasion through the inhibition of the PI3K/mTOR/MMP signaling pathway [151].

Furthermore, a detailed exploration of the underlying mechanism revealed that kaempferol treatment effectively hampers the activation of the transcription factor AP-1 and the MAPK signaling pathway. Moreover, kaempferol exerts its influence by suppressing the translocation of protein kinase Cδ (PKCδ) and the MAPK signaling pathway, consequently repressing phorbol-12-myristate-13- acetate (PMA)-induced MMP-9 expression and activity. Intriguingly, these results extend beyond the laboratory setting, as kaempferol has been shown to obstruct lung metastasis of B16F10 murine melanoma cells while simultaneously reducing the expression of MMP-9 in vivo. In summation, these findings collectively underscore the potential of kaempferol as a promising therapeutic candidate for combatting cancer metastasis [152]. In a study conducted by Qin and colleagues in 2016, they made significant observations regarding the impact of kaempferol on cholangiocarcinoma cell lines, specifically HCCC9810 and QBC939 [153]. Their research unveiled that kaempferol, administered at concentrations of 50 and 100 μM for 24 h, exhibited the ability to inhibit the migration and invasion of these cell lines. Furthermore, the study highlighted that kaempferol led to a reduction in MMP-2 activity, while concurrently promoting the expression of TIMP-2. Notably, there were no observed changes in the expression of MMP-9 and TIMP1. Additionally, kaempferol was found to exert its effects by inhibiting Akt and PI3K/Akt activation, shedding light on the underlying molecular mechanisms behind its inhibitory properties against cholangiocarcinoma cell migration and invasion [153].

### 5.4. Regulation of MMPs by Iso flavonoid genistein

Genistein, also known as 4′,5,7-trihydroxyisoflavone, is a naturally occurring compound abundant in soy-based products. Individuals who incorporate soy into their diet tend to have higher levels of genistein in their bloodstream compared to those following a Western-style diet centered around red meat. This difference in genistein levels has demonstrated varying effects in diverse clinical scenarios, including its impact on cholesterol regulation, osteoporosis, and cancer [154,155]. In the realm of oncology, genistein has garnered increased attention as a potential therapeutic agent. Population-based studies have highlighted the association between genistein consumption and a reduced risk of mortality from various cancer types, with notable emphasis on prostate and breast cancer [156,157]. This has spurred significant interest in exploring the potential benefits of genistein in the context of cancer prevention and treatment. The research conducted by the Shafiee group revealed that genistein possesses the capability to reduce MMP-2 expression across a spectrum of human prostate cancer cell lines, spanning from primary noncancerous cells to well-established metastatic variants [156].

Interestingly, little to no impact on MMP-9 was observed within this study. These effects were noticeable even at relatively low genistein concentrations, reaching as low as 10 nM. Furthermore, corroborating evidence of genisteins MMP-2 reduction has emerged from other scientific investigations, not only in prostate cancer but also in breast cancer and glioblastoma cell lines [158,159].

In the context of breast cancer, genistein treatment has been linked to decreased MMP-7 and MMP-9 expression [160]. Likewise, in vitro studies involving prostate cancer, glioblastoma, and pancreatic cancer have indicated a reduction in MMP-9 expression when exposed to genistein [161,162]. In the case of melanoma, breast cancer, and prostate cancer cells, genistein treatment results in a decrease in general zymographic activity, serving as a metric for MMP activity [162]. These collective findings illustrate that genistein exhibits the ability to selectively inhibit the production of specific MMPs. This property may have a substantial impact on a cancer cell’s capacity to invade and infiltrate the surrounding tissue. The effects of genistein on MMP-9 and TIMP-1, observed in in vitro studies, have also been substantiated by in vivo investigations using nude mouse xenografts populated with MDA-MB-231 and MCF-7 cells [159]. In these xenograft models, genistein exhibited the capacity to impede tumor growth, promote apoptosis, and enhance the expression of p21WAF1/CIP1 [163].

3D QSAR studies have substantiated the predictive capacity of genistein’s inhibitory activity against MMP1, MMP9, and MMP13. These predictions were derived from models established by taking into account the IC50 values for the compounds discussed in the article [164,165]. Genistein exhibits inhibitory effects on angiogenesis, both in in vitro and in vivo models [166]. By impeding the formation of new blood vessels, it disrupts the supply of vital nutrients to tumor cells and their connection to the circulatory system, thereby thwarting tumor metastasis. This intricate process is closely tied to alterations in the expression and activity of MMPs, highlighting the intricate interplay between c-erbB-2, NF-κB, MMPs, angiogenesis, basement membrane degradation, and tumor metastasis.

Increased MMP secretion by tumors is associated with lower apoptotic tendencies, reflecting the intricate regulation of MMPs and their inhibitors. Research findings underscore the multifaceted interactions between c-erbB-2, MMPs, telomerase, 143-3, Akt, and NF-κB DNA binding activity in influencing cell growth, differentiation, and apoptosis. Furthermore, this study reveals that genistein down-regulates c-erbB-2, suppresses the secretion of MMP-2 and MMP-9 in a dose- and time-dependent manner, and mitigates tumor cell invasion in the context of Head and Neck Squamous Cell Carcinoma (HNSCC), concurrently reducing the levels of 14-3-3 and phosphorylated Akt [167]. Xu and Daymond’s research demonstrates genistein’s ability to impede prostate cancer (PCa) cell invasion. This inhibitory effect is linked to MAP kinase, which plays a role in the activation of MMP-2 and cell invasion. Furthermore, genistein has the capacity to prevent the activation of p38 MAP kinase by TGFβ [168]. Genistein inhibitory effects on MMPs are attributed to its ability to modulate signaling pathways involved in cancer progression. Specifically, it has been demonstrated to downregulate the expression and activity of MMP-2 and MMP-9, key enzymes involved in extracellular matrix degradation and tumor invasion. By interfering with MMPs, genistein may play a significant role in suppressing cancer cell migration and metastasis.

The clinical significance of flavonoids in the context of MMPs in cancer holds considerable promise for advancing therapeutic strategies. Extensive preclinical studies have demonstrated the ability of flavonoids to modulate MMP expression and activity, pivotal factors in cancer progression and metastasis. The inhibitory effects of flavonoids on MMPs not only impede tumor invasion and angiogenesis but also exhibit potential in preventing the formation of distant metastases. Such findings suggest that flavonoids may have a clinically relevant role in mitigating the aggressive behavior of various cancers. Furthermore, the natural origin of flavonoids and their well-established safety profiles make them attractive candidates for integration into clinical interventions. As we transition from preclinical investigations to clinical trials, elucidating the specific mechanisms and optimizing dosage regimens, flavonoids may emerge as valuable adjuncts to conventional cancer therapies, offering a targeted and well-tolerated approach for improving patient outcomes.

## 6. Conclusion

In conclusion, the dysregulation of MMPs holds significant clinical implications in the context of cancer. Elevated MMP expression is often associated with tumor invasion, metastasis, and angiogenesis, making them crucial players in the complex landscape of cancer progression. Understanding the clinical relevance of MMPs opens avenues for targeted therapeutic interventions. Several MMP inhibitors are currently under investigation as potential anti-cancer agents, aiming to disrupt the intricate network of proteolytic activity that promotes tumor aggressiveness. While the potential for MMP-targeted therapies is promising, it is essential to acknowledge the existing limitations, including the complexity of MMP regulation and the need for precise targeting to avoid unintended consequences. Moreover, the heterogeneity of cancer types and individual patient responses underscores the importance of personalized approaches for effective MMP-based interventions.

Despite these challenges, ongoing research into MMPs provides valuable insights into their clinical significance, offering potential prognostic markers and therapeutic targets. By unraveling the intricate roles of MMPs in cancer progression, we pave the way for innovative strategies that harness this knowledge for improved patient outcomes. As we navigate the clinical landscape of MMPs in cancer, a nuanced understanding of their implications and limitations becomes paramount in shaping the future of precision oncology.

The multifaceted properties of flavonoids position them as promising candidates for the targeted regulation of cancer metastasis. Extensive research has revealed the potential of flavonoids in modulating both MMPs and EMT, pivotal processes in cancer metastatic cascade. The ability of flavonoids to inhibit MMP expression and activity contributes to their anti-metastatic effects by impeding crucial steps in tumor invasion and angiogenesis. Furthermore, flavonoids have demonstrated the capacity to interfere with EMT, inhibiting the transformation of cancer cells into a more invasive phenotype. This dual impact on MMPs and EMT underscores the comprehensive potential of flavonoids in disrupting the intricate molecular mechanisms driving cancer metastasis.

The exploration of flavonoids as agents targeting both MMP and EMT opens up avenues for the development of innovative therapeutic strategies against metastatic cancers. Their natural origin, coupled with demonstrated efficacy in preclinical studies, suggests that flavonoids may hold promise for clinical applications in cancer treatment. However, it is crucial to recognize the complexities of cancer biology and the need for further translational research to optimize the therapeutic potential of flavonoids. As we advance our understanding of these natural compounds, harnessing the synergistic effects on MMPs and EMT, we may unlock novel avenues for precision medicine approaches that combat cancer metastasis with greater specificity and efficacy.

## Figures and Tables

**Table 1:** Bioactive processes facilitated through MMP enzymatic cleavage.

	MMPs	Substrate cleaved	Biological Effect	Ref
**1**	MMP-1, 2, and 3	Fibronectin	Cell migration	[46–48]
**2**	MMP-2, MT1-MMP, MMP-19	Laminin 5γ2 chain	Epithelial cell migration	[11]
**3**	MMP-1, 3, 7 and –9	Processing IL-1β from the precursor, Pro-TNFα	Pro-inflammatory	[49,50]
**4**	MMP7	Heparin-binding EGF	Vasocontriction and cell growth	[51–53]
**5**	MMP7	RANK ligand	Osteocleast activation	[54]
**6**	MMP9	Galactin-3	Hypertrophic chondrocytes apoptosis and recruitment of osteoclast	[55,56]
**7**	MMP3	Plasminogen	Generation of angiostatin-like fragment	[57]
**8**	MMP3	E-cadherin	Epithelial-mesenchymal conversion	[58,59]
**9**	MMP2 and 7	Decorin	Increased bioavailability of TGF-β	[39,60]
**10**	MMP3 and MMP7	E-cadherin	Disrupted cell aggregation and increased cell invasion	[61,62]
**11**	MMP2	Chondroitinsulphate proteoglycan	Neurite outgrowth	[63]

## Data Availability

Not applicable.
